# Our Christmas Supplement

**Published:** 1902-12-13

**Authors:** 


### OUR CHRISTMAS SUPPLEMENT.

### THE HOSPITAL GARDEN.

### THE BEAUTIES OF EACH AND ALL THE HOSPITALS.

THE coronation year of King Edward VII. will be specially memorable in the history of the voluntary hospitals. In this year King Edward's Hospital Fund has for the first time distributed £100,000 amongst the metropolitan hospitals. This large sum, and the success which has attended the movement, have led the unthinking to rush to the conclusion, that this Fund will eventually receive the bulk of the money given each year to hospitals in voluntary contributions. This is of course an entire misapprehension of the objects of the King's Fund and of the situation generally, as it affects each hospital. When King Edward's Hospital Fund was founded it was shown, that collectively the metropolitan hospitals had an annual deficiency of quite £100,000 a year, when the total revenue of these institutions from all sources was compared with their actual expenditure.

The object of the King's Fund was to raise, in the main from entirely new sources, an additional revenue for the purposes of the London hospitals of at least £100,000 a year. The King, however, in his letter founding the Fund, mentioned £150,000 per annum as the maximum revenue which it would be the aim of the Fund ultimately to raise. There is now good reason to hope that this £150,000 will be forthcoming within the next few years, and that the King's Hospital Fund will then be maintained on that basis, probably for all time.

The completion of the work which the King's Fund was established to accomplish will, however, leave each London hospital where it has always stood in regard to the necessity, which every such institution must always labour under, of raising by its own individual efforts a large sum in voluntary contributions, to defray the cost of the greater portion of the work undertaken each year in the interests of the community. The voluntary system of hospital support, so far as this country is concerned, recognises the great underlying principle, that it is the right, indeed the privilege, of each citizen on his own initiative, as a mere matter of the highest duty, to render some measure of personal service to the sick. This principle is the bedrock upon which the support of our great English hospitals rests. The voluntary system has produced the best results in regard to administration, management, progress, and successful treatment within the hospitals which the world has so far attained to. The underlying reason for the establishment and success of King Edward's Hospital Fund, has been the recognition of this fact, and the conviction that the perpetuation of the voluntary system of hospital support is demanded in the interests of hospital efficiency, the adequate treatment of accidents and disease, and the well-being of the people at large.

In such circumstances the additional strength which has been given to the financial position of the voluntary hospitals in the metropolis, and indeed throughout the country, by the success of King Edward's Hospital Fund should stimulate individual effort on behalf of each hospital. It would indeed be a calamity, at a time when the burden of financing a single hospital has been permanently relieved by the co-operation of the King's Fund, for the managers of such an institution to slacken their efforts, instead of being stimulated to greater exertions by the generous assistance received from the great central fund. The hope and wish are to perpetuate and develop individual effort, and we have so much confidence in the intelligence of the army of good men and women who are at present responsible for the efficiency of our voluntary hospitals, that we feel certain this result, at any rate, must ensue from the marked success of King Edward's Hospital Fund.

Before the days of the King's Fund it was often difficult for the managers of a particular hospital to persevere in their efforts, much less to increase them, when each year proved that the claims upon an Institution's resources showed a steady increase, whilst the contributions of the benevolent to it did not grow in anything like the same proportion. The King's Fund has altered all this by diminishing the burden of outstanding indebtedness and giving to many of the managers a guarantee of a definite yearly sum, sufficient to enable them to work their hospital at its full strength, and so upon the most economical basis. Thus the King's Fund has so far proved a double blessing, for, whilst it has increased both the permanent and casual revenues of the hospitals, it has tended to diminish the cost of their maintenance by placing the managers in the best position to increase efficiency at the smallest expenditure compatible with the excellent results achieved.

It follows that the collective efforts represented by the central fund do but everywhere enforce the necessity for continuous individual effort by those immediately responsible for each particular hospital. We have thought that our Christmas Number this year might be made helpful in enforcing this view if we were to treat the whole of the hospitals as a bouquet of benevolence, from which those who are interested in these institutions may select the particular flower which offers them the highest attraction. As the central funds have immensely deepened and extended the interest which the population at large now feel in the hospitals, and have greatly increased the area of givers to the medical charities, so we hope that the advent of this army of new givers, through the central funds, may be made the occasion for individual hospitals going into the field and reaping the best results for their own institution. With the object of rendering this development in a new direction as successful as possible, we have spared no pains to collect accurate data to enable us to attractively describe each particular flower as represented by one hospital and its work. That work will be briefly indicated, and we shall set forth the present needs and claims of each institution, so that every reader may readily obtain a bird's-eye view of the whole hospital garden. He can then select of his own free choice that portion to which he will particularly devote himself, not only during 1903, but for the future. We cannot help feeling that a perusal of the many interesting and novel facts which we have collected, must possess an attraction which should lessen the labour of securing new contributors to individual hospitals. Our readers will realise, as we do, the great indebtedness which the whole community owes to the permanent officials of our hospitals, who in good, and still more in bad times, continuously labour, despite great and ever-increasing difficulties, to secure a sufficient amount of monetary support for their hospitals. Such pecuniary aid is essential to enable the managers to deal adequately with the mass of human suffering and misery which it is the privilege and pride of our voluntary hospitals to successfully relieve each year. Nowadays hospitals are the fashion, and we hope that 1903 may prove to be the most fruitful and successful year which the London hospitals have yet experienced in their splendid history.

### WHAT EACH HOSPITAL NEEDS AT THE MOMENT.

IN pursuance of our plan of taking the bouquet of charity as represented by the London hospitals, and dealing with each flower or hospital in order, we shall endeavour to bring out clearly what each hospital is doing, what its present financial position is, and what are its most pressing requirements, which a benevolent public can advantageously further by a contribution in cash. We propose to take the metropolitan hospitals in the order of their size, and to facilitate a reference we shall include at the end an alphabetical list with the page on which each institution appears. We hope that the large number of wealthy people who have made it a practice to preserve the Christmas Appeal Number of THE HOSPITAL for reference throughout the year may be considerably increased this Christmas, and that all who use this issue as a handy book of reference for the purposes of their charitable gifts will find the plan pursued this year so helpful as to induce them to contribute even more liberally than they have heretofore done to the relief of sickness and accident in the metropolis of the Empire.

The Distribution Committee of the Hospital Sunday Fund has always had behind it the full confidence of the public, and we have therefore decided to quote the figures from the particulars issued under its authority. It is generally recognised that the great general hospitals are the backbone of our hospital system, and the following striking figures give (1) the number of beds, (2) the average number of beds daily occupied, and (3) the number of in-patients under treatment during 1901 in the greater hospitals of London—that is, those containing 150 beds and upwards.

| Name of Hospital. | No. of Beds. | Average No. of Beds daily Occupied. | No. of In-Patients Treated. |
|---|---|---|---|
| London Hospital ... ... | 790 | 678 | 12,723 |
| St. Bartholomew's Hospital ... | 741 | — | 6,093 |
| Guy's Hospital ... ... ... | 652 | 469 | 7,326 |
| St. Thomas's Hospital ... ... | 586 | 474 | 6,605 |
| Middlesex Hospital ... ... | 381 | 297 | 3,777 |
| St. George's Hospital ... ... | 351 | 300 | 4,448 |
| Hospital for Consumption, Brompton ... ... ... | 321 | 248 | 1,241 |
| St. Mary's Hospital ... ... | 281 | 250 | 3,812 |
| Dreadnought (Seamen's) Hospital ... ... ... ... | 269 | 236 | 2,[illegible]54 |
| Hospital for Sick Children ... | 232 | 172 | 2,314 |
| King's College Hospital ... | 223 | 184 | 2,582 |
| Westminster Hospital ... ... | 213 | 179 | 2,743 |
| National Hospital for Paralysed | 200 | 177 | 952 |
| University College Hospital ... | 185 | 164 | 2,695 |
| Royal Free Hospital ... ... | 170 | 13[illegible] | 2,026 |
| City of London Hospital for Diseases of the Chest ... | 164 | [illegible]20 | 966 |
| Great Northern Central Hospital | 159 | [illegible]34 | 1,811 |
| Royal National Hospital for Consumption ... ... ... | 152 | 144 | 776 |
| Charing Cross Hospital... ... | 150 | 100 | 1,584 |

The figures which are given above indicate that the London Hospital passes nearly twice as many in-patients through its beds as any other great hospital. What is the explanation of this? Are the patients discharged to a convalescent institution, or how is it managed?

### London Hospitals with 150 Beds and Upwards.

### THE LONDON HOSPITAL.

This hospital heads the list, because it is the largest of our hospitals, and passes through its wards each year more than double the number of in-patients treated by any other metropolitan hospital with the exception of Guy's and St. Thomas's. The number of out-patients, casualties and all other cases treated at the London during 1901 was upwards of 169,000. It is situated in the East End, where its influence for good is not confined to the medical relief it affords, for the humanising and civilising influences which result to a patient from a residence within its walls are of immense value to the citizens by raising the character of large numbers of the population throughout one of the poorest districts of London. This may also be said with truth in regard to other hospitals, but the influence in the case of the London is patent and appreciable owing to the vast numbers handled every year. Each day the hospital expends £240 in relief, and the annual expenditure now amounts to about £87,000. It is estimated that this expenditure must increase to £100,000 a year when the rebuilding and reconstruction now proceeding are completed. About one-fourth of the latter sum, or say £25,000 a year, is derived from invested funds, and the balance of £62,000 has to be raised from the public. During the last three years the average gross annual income has been £98,398, and the average gross annual expenditure £112,231, showing a deficiency of about £14,000. Mr. Sydney Holland, the indefatigable chairman, has exhibited much zeal for this hospital, and has been very successful in raising money for it. The most pressing requirements are funds for the rebuilding of the hospital, which is now proceeding, at an estimated cost of £370,000. Of this, £202,421 has been spent, and there remains £167,000 to be found before the whole work can be finished, unless the hospital is to further reduce its invested funds, which are none too large, considering the magnitude of its responsibility. An isolation block has been built, costing £30,000, five operation theatres have been provided at a cost of £13,000, a new out-patient department has been erected, costing £70,000, including accommodation for such special departments as throat and ear, eye, dental, electrical and light treatment, and massage. A pathological department with two post-mortem theatres has been erected at a cost of £19,000, and the balance of the £202,000 odd has been expended in replacing or renewing the old buildings which contain the wards. A recent visit to the London showed that the present house-governor, Mr. G. Q. Roberts, has complete touch with everything that is going on in regard to the extension and building works, the whole of which he seems to have at his fingers' ends, a fact which may well give confidence to wealthy people who can afford to give large sums to London's largest hospital. It would be a calamity to East London if the work of this noble institution had to be curtailed. Its present circumstances justify special help, and everybody will have an opportunity, which we hope they will take, of contributing to its funds during 1903, when a special appeal will be issued for its Five Years Sustentation Fund.

### ST. BARTHOLOMEW'S HOSPITAL.

This is the only hospital in the country which is able to meet the whole of its expenditure out of the revenues derived from its invested property and assets. It has just acquired about an acre of ground from the trustees of Christ's Hospital, at a cost of about £250,000—a sum that everybody hopes it will provide out of its vast resources—of which the governors may well be proud. It would, indeed, be little short of a calamity, if this great institution were to be allowed to come down from its position of splendid isolation as the one independent hospital, where the management of its assets and properties has heretofore been so excellent, that within living memory it has never issued an appeal to the public for funds.

### GUY'S HOSPITAL.

Guy's Hospital has always and deservedly held a high position on account of the great skill and reputation of its medical and surgical staff. It has made great strides during recent years under the able administration of Dr. Perry, the medical superintendent, and its financial position has been materially strengthened through the devoted energy and influence of the treasurer, Mr. H. Cosmo Bonsor. The old parts of the building are being gradually renewed and modernised, a new nursing home of magnificent proportions (the gift of a benevolent donor) has recently been opened, the condition of the wards has been greatly improved, and Guy's renders an immense service to the Metropolis by the relief which it affords to the inhabitants of the Borough and to residents in South London especially. Its financial position is steadily improving. During the last three years the average gross annual income has been £73,730, and the average gross annual expenditure £88,356, showing a deficiency of upwards of £14,000. The pressing requirements of the hospital are: (1) money to discharge debt, and (2) increased income to enable the governors to keep open all the wards. Guy's badly needs further money to modernise several portions of the old buildings, and to provide new operation-theatres which shall contain every modern requisite. Benevolent donors who, like Mr. Carnegie, believe in the encouragement of thrift, should take Guy's Hospital under their especial patronage, because the governors have had the courage to maintain pay wards, which have rendered great service to many deserving people. In this matter of pay wards Guy's Hospital has set an example to all the general hospitals which they might follow with advantage to the whole community. It would redound to their credit if the managers of every great hospital, where it is possible for them to provide a site upon which a separate pay wing can be erected, were to make ample provision for the reception of paying patients.

### ST. THOMAS'S HOSPITAL.

This is one of the best known of our hospitals, owing to its situation on the Albert Embankment, where it occupies one of the most splendid sites in London. Six thousand six hundred and five in-patients and 62,681 out-patients were admitted for treatment during last year. During the last three years the average gross annual income amounted to £64,451, and the average gross annual expenditure to £67,743, showing a deficiency of upwards of £3,000, to meet which contributions will be gratefully received by the treasurer, Mr. J. G. Wainwright, who for a large number of years has rendered yeoman service to the institution. The present requirements of St. Thomas's Hospital are: (1) certain reconstruction works connected with the in and out-patient departments, (2) a new nurses' home, and (3) a laundry. The estimated cost of all this is about £100,000. Contributions to the reconstruction fund will be gladly accepted, especially as some misapprehension appears to have arisen in regard to a recent legacy which will not, we understand, be paid for some time to come.

### MIDDLESEX HOSPITAL.

THIS instituion must always commend itself to the support of the charitable owing to its cancer wards, which have done an immensity of service to an army of sufferers, and which are so admirably administered as to constitute probably the best provision for cancer to be found anywhere in the world. In 1891, in addition to the in-patients, 45,084 out-patients were admitted to treatment. During the last three years the average gross annual income has been £36,884, and the average gross annual expenditure £38,363, showing a deficiency of about £1,500. Of the legacies, etc., received, upwards of £3,000 has been invested on an average each year. Certain important building improvements, including a new laundry and nurses' home, are well in hand, and wealthy scientists may be glad of an opportunity of contributing liberally towards the Cancer Research Fund at this hospital. This hospital has the distinction of retaining the life-long service of its principal officers. The pleasant personality of the secretary, Mr. F. Clare Melhado, makes visitors feel at their ease and so in the best humour to give generously.

### ST. GEORGE'S HOSPITAL.

THIS institution renders great service to the wealthier residents as well as to the poor in the district which it serves. In addition to the in-patients, it relieved last year 31,843 out-patients. During the last three years the average gross annual income has been £34,195, and the average gross annual expenditure £45,602, showing a deficiency of upwards of £12,000. Its pressing requirements are: (1) a considerable expenditure upon the buildings and equipment of the hospital, and (2) the awakening of the wealthy residents around the hospital to the fact that its income is too small for the work it annually does, to so large an extent in their especial interest. Formerly St. George's Hospital had the finest revenue from annual subscriptions of any London hospital, but year by year there has been a gradual diminution in this important source of income, which would be unaccountable and which we make bold to say would not even exist, if the subscriptions had been given by the relatively poorer residents to a hospital in a crowded district of London. St. George's Hospital occupies the most conspicuous site of any London hospital, a fact which, when taken in connection with the gradual falling off in the revenue from annual subscriptions, must be accepted as an indication that the popular belief that it is desirable, for financial reasons, that the site of a big hospital shall be in a leading thoroughfare, is largely erroneous. Looking at the circumstance that the annual subscription-lists of almost every other voluntary hospital in London are steadily increasing in amount and length, we are moved to urge the wealthy residents around St. George's Hospital, and the wealthy frequenters of Hyde Park and the Row, in defence of their own reputation and credit, to make it their business to send each year a definite contribution to this important institution. Mr. Timothy Holmes, the Treasurer, and Mr. C. L. Todd, the Secretary, are indefatigable in their labours on behalf of St. George's Hospital.

### THE BIRMINGHAM GENERAL HOSPITAL.

THIS hospital was erected and opened in 1897, and has one of the most attractive and striking elevations of any hospital in the country. It is administered by a committee which is without doubt one of the most active and capable bodies charged with the management of a great hospital. It should have an immense interest for musical people of high and low degree, for the Birmingham Festival had its origin in, and owes its success to, the circumstance that it is held every three years in aid of this great medical charity. We have never agreed with, and a recent inspection has confirmed our objection to, the system which prevails whereby the whole establishment, including the quarters of the resident medical officers and staff, is ventilated by artificial means only. However, the methods pursued are still in the experimental stage, and we have only mentioned them because they constitute an interesting feature of this particular hospital, which is well worthy of a visit. The hospital contains 346 beds, of which 278 were daily occupied during last year, when 5,170 in-patients and 62,642 out-patients were relieved. In 1901 the total income from all sources was £26,681, and the total expenditure £25,224. This shows an apparent surplus of upwards of £1,400. We say apparent, because a sum of £5,105, the proceeds of the Hospital Sunday collections which are only received once in three years, is included in the total. There was in addition a deficit of £6,291 brought forward from the previous year. The most pressing requirement of this hospital is a considerable increase in the income to maintain the institution in full working order, and to meet the large and increasing demands made upon it year by year. Few if any provincial hospitals are as well administered as the Birmingham General Hospital, and it is fortunate to have as its house-governor and secretary Mr. Howard J. Collins, who has had considerable experience in the working of London and provincial hospitals, and is deservedly popular with the Committee and the Governors.

### THE HOSPITAL FOR CONSUMPTION, BROMPTON.

PHTHISIS is the curse of our climate, and all the consumption hospitals, and especially that at Brompton, should receive the liberal support of all thoughtful people. It is probable that no institution has a greater or more pressing demand upon its beds than the Brompton Hospital. It admitted 7,124 out-patients to treatment during last year. For the last three years the average gross annual income was £31,734, and the average gross annual expenditure £34,281, showing a deficiency of about £2,500. Its special requirements are: (1) gifts in money to discharge this deficiency, and (2) more liberal support from living contributors, who might well follow the splendid example set them by deceased benefactors, for the contributions by legacy to this hospital are so large as to be distinctly remarkable. Secretary, Mr. W. H. Theobald.

### ST. MARY'S HOSPITAL.

THIS hospital ministers to the needs of the poor scattered over the large area included in Paddington and the neighbouring districts. Last year it relieved 43,681 out and midwifery patients in addition to upwards of 3,800 in-patients. It has always enjoyed a wide popularity, but has lacked the spontaneous support of not a few of the many wealthy people who reside within the area which it serves. During the last three years the average gross annual income has been £24,837, and the average gross expenditure £29,534, showing a deficiency of upwards of £5,000 per annum. In order to better supply the needs of the district, new buildings are in course of erection which will cost about £50,000. This money has been provided largely through the energy of the secretary, Mr. Thomas Ryan, who deservedly received the special acknowledgments of the Governors, which were prominently recorded in the annual report. The income to meet the increased cost of maintaining the extra beds and upkeep has yet to be raised. The present requirements of this hospital are: (1) additional annual subscriptions and a larger revenue for general purposes; (2) an adequate nursing home which is essential to the proper administration of the hospital. There must be many wealthy people in Paddington who should be glad of the opportunity to defray the whole cost of the home for nurses, as was done by a Governor of Guy's where the Raphael Home was recently opened by H.R.H. the Prince of Wales.

### DREADNOUGHT (SEAMEN'S) HOSPITAL.

FREE to seamen of all nations, regardless of creed, race, or colour, this hospital appeals to everybody for support. In recent years it has added to its claims excellent provision for the reception of consumptives, who are now treated on the open-air principle. It has further rendered the Empire material service by erecting and maintaining the School for Tropical Diseases, and a new hospital in the East and West India Docks. Mr. P. A. Nairne, the chairman, has devoted the greater part of his life to this hospital's interests, and Mr. P. I. Michelli is one of the most enthusiastic and indefatigable of secretaries. With such claims the Dreadnought Hospital must appeal to everybody who has money to give in charity. Last year it treated 22,542 out-patients in addition to 2,634 in-patients. During the last three years the average gross annual income has been £16,963, and the average gross annual expenditure £20,071, showing a deficiency of upwarcs of £3,000 per annum. Its most pressing requirements are: (1) £10,000 to provide new flooring and furniture for all the wards, dining-halls and day-rooms, and the provision of electric light and many structural improvements; (2) an additional income from annual subscriptions of £5,000 per annum. The requirements of this hospital are so relatively modest that, in view of the claims it undoubtedly has upon the whole population, they should be immediately supplied.

### HOSPITAL FOR SICK CHILDREN, GREAT ORMOND STREET.

THIS is probably the oldest children's hospital, and to its successful development we owe most of the children's hospitals in the country. It has rendered great services to the poor of the metropolis. Last year it relieved 24,670 out-patients and 2,314 in-patients. During the last three years its average gross annual income has been £18,957, and its average gross annual expenditure £19,689, showing an annual deficiency of about £750. The number of patients benefited shows an alarming tendency to grow every year. Its especial needs are: (1) more income, and (2) a large sum to enable it to procure a site and erect an out-patients' department of ample proportions, replete with every modern convenience. It has the advantage of having on its Committee many men of high public reputation and ability, and its secretary, Mr. Adrian Hope, is one of the most active and successful of hospital secretaries.

### KING'S COLLEGE HOSPITAL.

THIS hospital is erected upon an old burial ground, and the site has never been quite satisfactory. At the present time a new out-patients' department is urgently called for, but an adequate site is not forthcoming. Much might be urged in favour of the removal of this hospital to the suburbs were it not for its affiliation to King's College, which has so far prevented, and will probably always prevent, any step being taken in this direction. It relieved 20,471 out-patients and 2,582 in-patients last year. It has immense claims upon the public from the circumstance that it is Lord Lister's hospital, and to Lord Lister all those who are surgically sick owe more probably than to any other living man. During the last three years the average gross annual income has been £19,698, and the average gross annual expenditure £19,625. Here, then, is a hospital economically administered with a strict regard to the provision of funds before liability is incurred, a policy which should win for King's College Hospital the generous recognition of all business men and practical philanthropists. Its most pressing requirements are (1) a new out-patients' department, (2) a new nurses' home, and (3) the reconstruction of the lavatory and bath accommodation, together with other structural alterations, for which purpose a special appeal is about to be issued for £75,000. This money ought to be speedily forthcoming, for King's College Hospital is exceptionally well administered. It has direct claims upon every churchman and every member of the legal profession, and we believe its requirements have only to become sufficiently known to ensure the receipt of all the money needed. Secretary, Mr. H. S. Tunnard.

### WESTMINSTER HOSPITAL.

THE claims of Westminster Hospital are based first of all upon the fact that it has always been administered with exceptional economy. Mr. Sidney M.

Quennell, the Secretary, is one of the oldest and deservedly one of the most trusted and respected of hospital officials. The success which attended the special appeal during 1901 should encourage the committee to impress their needs and claims continuously upon the many wealthy and well-to-do residents in Westminster. It relieved 22,290 out-patients in addition to 2,743 in-patients last year. During the last three years the average gross annual income has been £24,811, and the average gross annual expenditure £21,595, showing an apparent surplus of rather over £3,000 a year. The special requirements of the hospital are: (1) additional funds to enable pressing structural changes to be made, including a bath-room for the obstetric ward, a new casualty room, properly lighted, so as to make it available for minor operations, and the reconstruction of the sanitary arrangements connected with some of the ward kitchens and w.c.'s.; (2) a nursing home for the accommodation of the nurses at this hospital would be an immense gain, as it would give certain much needed extra accommodation within the hospital proper, and so add to the comfort and efficiency of the whole establishment. It is surely not too much to ask of the residents of Westminster to subscribe £50,000 without delay to enable all these important matters to be taken in hand.

### NATIONAL HOSPITAL FOR THE PARALYSED.

This hospital is slowly recovering from the effects of the prolonged discussions which recently took place with regard to its administration. It is an essential part of the hospital system in the metropolis, and in these days of hurry and strain every worker who has the means should make a point of giving something of his abundance in support of the National Hospital for the Paralysed. It relieved 6,150 out-patients and 950 in-patients last year. The fact that with 200 beds only 952 in-patients should have been admitted for treatment emphasises the special character and difficulty of the work done by this Institution for the public of all classes. During the last three years the average gross annual income has been £13,174, and the average gross annual expenditure £16,145, showing a deficiency of about £3,000 per annum, for which an urgent appeal is made. The pressing requirements of this hospital include: (1) additional income, (2) the provision of £10,000 to enable the committee to take in hand the redecoration of the whole Institution, certain necessary sanitary works, and the provision of more ample and suitable accommodation for the nurses and servants of the establishment. We hope the whole of this £10,000 may be forthcoming without delay, as it most certainly would be, did the wealthier classes realise what the work of this hospital means for everybody and how pressing are its claims upon their generosity.

### UNIVERSITY COLLEGE HOSPITAL.

This institution has never attracted the financial support which its position as the hospital of University College ought to have secured for it long ago. It has the advantage of a new building (the generous gift of Sir J. Blundell Maple), now in course of erection on a somewhat novel plan, which is full of interest to the student of hospital construction. So soon as the whole of the old buildings have been demolished and the new hospital completed and opened, many thousands of pounds will be required for upkeep. Forty-two thousand five hundred and sixty-nine out-patients and 2,695 in-patients were admitted to treatment last year. During the last three years the average gross annual income has been £22,973, and the average gross annual expenditure £20,968, showing an apparent surplus of nearly £2,000 a year; we say apparent because, had the hospital not been in course of rebuilding, there would not only have been no surplus but a very great deficiency. The pressing requirements of this hospital this year are: (1) money to defray the cost of furnishing the new buildings; and (2) an additional income of £5,000 per annum at least. Secretary, Mr. Newton H. Nixon.

### ROYAL FREE HOSPITAL.

The Royal Free Hospital has special claims upon the community as the first general hospital to which all classes of patients have from the outset been admitted free and without tickets. The example which it set at a time when the ticket system was rampant has yielded excellent fruit, for to-day, with a very few exceptions, the chief passport to admission at the great London hospitals is, the urgency of the medical or surgical needs of each patient who presents himself for treatment. The Royal Free Hospital relieved 35,409 out and midwifery patients and 2,026 in-patients last year. During the last three years its average gross annual income has been £12,163, and its average gross annual expenditure £12,176. These figures afford the best proof of the economy and wise financial management of this hospital under the able guidance of Mr. Charles Burt, the chairman, who has worked most successfully and zealously on its behalf, and has been most ably seconded by Mr. Conrad W. Thies the secretary. The pressing requirements of the hospital are: (1) structural alterations and the provision of new and improved sanitary appliances and baths; (2) a new out-patient department; and (3) new floors throughout the hospital. An immediate gift of £10,000 and a considerable influx of annual subscribers are needed for this most deserving and excellent hospital.

### CITY OF LONDON CHEST HOSPITAL, VICTORIA PARK.

This is the Hospital in which the late Dr. Peacock, the eminent heart specialist, took a life-long interest. His memory must still be dear to very many wealthy families, and to them, and indeed to all, we can cordially commend the claims of this useful institution. Last year it relieved 9,678 out-patients and 966 in-patients. Owing to lack of funds, a few years ago a very considerable number of the beds had to be closed. The committee took vigorous measures to improve the financial position, and at the present time they have succeeded in re-opening the majority of the beds. During the last three years the average gross income has been £12,280 and the average gross annual expenditure £11,314. The small surplus thus shown is more apparent than real, and is due, we believe, mainly to the laudable desire of the committee to keep out of debt. The invested funds equal less than half of the mortgage debt which it is desired to pay off as speedily as possible. The most pressing requirement of the hospital is: (1) a new nurses' home, and having regard to the fact that consumption is a disease which unfortunately finds a place in the majority of families, we have great confidence that the fact has only to be stated, to insure that some generous contributor will come forward and erect a new home for the nurses as a memorial, or as a thank-offering for the recovery of some dear friend or relative. The secretary, Mr. Henry T. Dudley-Ryder, is entitled to much credit for the improvement which has taken place in the financial position since he assumed office.

### GREAT NORTHERN CENTRAL HOSPITAL, ISLINGTON.

At a time when some are agitating for the removal of certain hospitals from their present sites to the suburbs, the Great Northern Central Hospital should attract deep interest and secure abundant support. The Great Northern Hospital was moved from King's Cross to its present site in 1887, and secured incorporation by Royal Charter in 1900. The change of site induced the inhabitants of North London to come forward and subscribe sufficient funds to defray the whole cost of the buildings of the new hospital, and left a balance sufficient to enable the committee to invest some £30,000 as the nucleus of a reserve fund. It relieved 26,632 out-patients and 1,811 in-patients last year. It has rectangular and circular wards—a plan of construction which has enabled the fullest advantage to be taken of a somewhat restricted and difficult site. It is admirably administered, and has the advantage of the services of Mr. Lewis H. Glenton-Kerr, the secretary, whose successful work has earned for him an enviable reputation. During the last three years the average gross annual income has been £12,002, and the average gross annual expenditure £13,022, showing a deficiency of about £1,000 a year. Its most pressing requirement is additional income, and all who take an interest in modern hospitals should visit this institution, after which we are confident they will become permanent and liberal subscribers.

### ROYAL NATIONAL HOSPITAL FOR CONSUMPTION.

This hospital consists of a number of separate houses or villas erected at Ventnor, Isle of Wight. It is conducted on what is known as the separate or home system, and has been widely popular in consequence. There were no out-patients, but the number of in-patients admitted last year was 776. During the last three years the average gross annual income was £18,327, and the average gross annual expenditure £17,285, showing a surplus of £1,100 a year. So long as consumption continues to prevail, as we fear it must always prevail in our climate, so long will this institution continue to receive, as it deserves, liberal support from the public.

### CHARING CROSS HOSPITAL.

This institution has often been described as the central hospital for accidents in London. It has undergone considerable structural alteration, and a large sum is at present being expended upon new buildings. It occupies the chief portion of a site isolated by streets off the Strand near Charing Cross, and for years public opinion has demanded that it should acquire the whole of this self-contained site, including that portion of it which is at present occupied by the Royal Westminster Ophthalmic Hospital. It relieved 13,820 out and midwifery patients, and 1,584 in-patients last year. During the last three years the average gross income has been £25,508, and the average gross annual expenditure £20,609. This apparent surplus of £5,508 per annum represents the additional contributions which have been raised towards the special reconstruction fund, and at the present time this hospital is appealing for £50,000 to make up the sum required to complete the rebuilding. The hospital is at the present time in a state of transition, but its most pressing requirements are: (1) an additional £50,000 for the rebuilding fund, and (2) an increase of £5,000 a year to its income from voluntary sources. Mr. Arthur Read, the secretary, who has long been connected with the institution, is one of the most popular personalities in the world of hospital officials.

### HOSPITALS WITH 100 BEDS AND UPWARDS.

### LONDON FEVER HOSPITAL,

Liverpool Road, Islington, N.

This is one of the most important and useful of London hospitals. For a century at least it has rendered incalculable service to middle-class families by admitting children and adults suffering from fever, the majority of whom pay something towards the cost of their treatment. It contains 200 beds, but the average number occupied necessarily varies considerably from year to year. Last year 83 beds were daily occupied, and 810 in-patients were admitted to treatment. During the last three years the average gross annual income was £13,136 and the average gross annual expenditure £12,539. In busy years, when fever is rampant in London, the expenditure often largely exceeds the revenue. The pressing requirements of this hospital are (1) about £10,000 to defray the cost of reconstruction and of rebuilding the older portions of the hospital, which it is essential should be completed without a moment's avoidable delay; and (2) a convalescent home where infectious convalescents can be taken and isolated. This last would be a boon to Londoners, and it is eloquent testimony to the want of apprehension and grasp displayed by the average well-to-do resident in the metropolis of the Empire, that funds for this convalescent branch are yet to seek. The efficiency of the hospital is proved by the fact that there is probably not a medical man in London who, when he contracts fever himself, or has it in his own household, does not use every means to secure admission for himself or the members of his family to this institution. The president, Lord Balfour of Burleigh, takes an active interest in the work of the hospital, and under the present secretary, Major W. Christie, the institution has prospered and progressed.

### ROYAL SEA-BATHING HOSPITAL,

### Margate.

This institution, situated at Margate, has, for some reason which it would be difficult to explain, never yet attained the position which it ought to occupy as the largest special institution for the treatment of scrofula and kindred diseases. Somehow it has failed to secure the continuous efficiency in its administration which is so marked a characteristic of larger voluntary hospitals. There is a lack of concentrated energy and progressive growth in its revenues and extension which is lamentable, in view of the undoubted necessity which exists for its efficient maintenance. The main reason possibly is that its situation at Margate keeps it largely out of sight, and so out of mind, in the case of the great hospital public. One hundred and twenty of its 150 beds were daily occupied last year, and 651 in-patients were admitted to treatment. During the last three years the average gross annual income has been £12,199, and the average gross annual expenditure £8,430. It is very greatly in need of money for the furtherance of its useful work. Mr. Michael Biddulph, the treasurer, has devoted much time and energy to this hospital, and his devotion ought to attract a number of active and capable men to the management, when we have no doubt the work of the Royal Sea bathing Hospital would extend and multiply exceedingly.

### ROYAL LONDON OPHTHALMIC HOSPITAL.

This hospital has suffered especially from the diversion of funds through the war. In 1899, when the new building was occupied, the committee intended to appeal to the public for increased support to meet the necessary increased expenditure consequent upon the occupation of a larger and modern building. The war began at that very time, and, so far from obtaining increased support, it has been difficult to collect the usual subscriptions and donations. The hospital has no more investments which can be realised, and therefore the committee earnestly appeal for generous help. This is no ordinary application for help; it is the earnest appeal of the managers of a great charity which has occupied an unique position in the kingdom for nearly a century, and which is now passing through a period of exceptional strain—a critical period which must be a turning point in the history of the oldest and largest eye hospital in England. Secretary, Mr. Robert G. Bland, City Road, E.C.

### EAST LONDON HOSPITAL FOR CHILDREN.

Any person of means who takes a real interest in the institutions of the London should make it their business on a wet, cold, miserable day to drive to Glamis Road, Shadwell, and call on the matron or secretary of the East London Hospital for Children. For thirty years we have been inspecting hospitals of various kinds in all parts of the world, but we can truthfully say that every time we enter the East London Hospital for Children we are more and more impressed with the quality of the work it is doing for East London. On each occasion of our inspection we have been struck with the circumstance that the children, though many of them are quite infants, are invariably quiet and happy. There is a freedom from the distressing wailing which is often met with in children's hospitals, and we take this fact to be the best evidence of the excellence of the administration of every department of this institution. To pass out from the gloom and squalor of the streets on such a day as we have indicated into the wards of this hospital is an altogether encouraging and surprising pleasure which nobody should miss. Of its 135 beds 117 were daily occupied last year. It relieved 2,192 in-patients and 36,475 out-patients. During the last three years the average gross annual income has been £10,466, and the average gross annual expenditure £10,122, a proof of the economy and care with which the expenditure is controlled. Its most pressing requirements are (1) an isolation ward; (2) a nursing home; and (3) certain structural alterations. We hope that before long money for all three will be provided. Mr. Thomas Hayes has held the post of secretary for a good many years, and the institution has prospered and extended under his management.

### LONDON LOCK HOSPITAL AND RESCUE HOME.

Help is required for this valuable institution. There has been a continual flow of patients to the Hospital, some of whom have found their way to the Home and other homes, in many cases with very good results. It is the desire of all connected with the Hospital and Home to do the utmost in their power to succour those who are in want of a helping hand. Secretary, Mr. A. W. Cruickshank.

### WEST LONDON HOSPITAL,

### Hammersmith.

This hospital has added much to its claims upon the liberal support of the charitable public by throwing open its wards and organising a school for post-graduates. This step is calculated to gradually increase the usefulness and efficiency of the whole institution. Of its 133 beds 123 were daily occupied last year. It relieved 2,126 in-patients and 32,176 out-patients. During the last three years the average gross annual income has been £9,737, and the average gross annual expenditure £9,434. If the committee has a fault we should say it consisted in timidity, due to the enforcement of the strictest economy and care in regard to expenditure. This is a good fault, no doubt, and in view of the encouragement and assistance which this hospital has received of late, we believe the whole institution will speedily be brought up to a high state of efficiency. On a recent visit we were much struck with the knowledge and earnest interest displayed by the secretary, Mr. R. J. Gilbert. The most pressing requirements of this institution are: (1) a new nursing home, for which plans have been prepared; (2) £30,000 to enable portions of the hospital to be reconstructed, and an administration block with additional wards built; (3) an addition to the present site, owing to the circumstance that it would be unwise, and indeed wrong, to attempt to put on the present restricted site all the buildings shown on the completed scheme given in the plan in the annual report. This hospital has made considerable strides of late. It ministers to a large and increasing population, and should receive a much greater measure of support from the public in the district which it serves.

### LONDON TEMPERANCE HOSPITAL.

THIS hospital has greatly improved in every respect in recent years, and is now one of the best planned and most comfortable hospitals in the metropolis. Of its 120 beds, 84 were constantly occupied last year. It relieved 1,299 in-patients and 21,853 out-patients. During the last three years the average gross annual income has been £19,675, and the average gross annual expenditure £11,418. Its pressing requirements are: (1) £15,000 for a new out-patient department, which is badly needed. Having regard to the spread of temperance principles throughout the nation, we should hope that this money will be forthcoming without delay or difficulty now that the need is recognised and the facts are known. Secretary, Mr. A. W. Bodger.

### METROPOLITAN HOSPITAL.

THIS institution is situated in one of the poorest and most crowded districts of London. It deserves a much larger measure of support than it has heretofore succeeded in attracting. Of its 111 beds 86 were daily occupied during last year, and it relieved 1,131 in-patients and 39,431 out-patients. During the last three years the average gross annual income was £11,624, and the average gross annual expenditure £11,066. Its most pressing requirements are: (1) Some £20,000 for a nurses' home which is badly needed, in order to free for the reception of patients the wards now converted into cubicles. Its secretary, Mr. Chas. H. Byers, has for many years laboured assiduously and successfully to extend the resources of this important hospital, which in the past has been far too little known to wealthy contributors to hospitals—a disadvantage which we hope will speedily disappear.

### CANCER HOSPITAL.

THIS hospital was founded in 1851 for the free treatment of the necessitous poor who are afflicted with cancer, tumours, or allied diseases. The nature of the disease makes it necessary to supply both food and dressings in great quantity and of high quality. The annual expenditure amounts to about £12,000, to meet which there are annual subscriptions of £2,000 and dividends £3,400. The balance has to be made up by donations, etc., and generally it means selling out stock to the amount of about £3,000 or £4,000. Secretary, Mr. F. W. Howell.

### LONDON HOMŒOPATHIC HOSPITAL.

A GREAT work is carried on by this institution, and one that undoubtedly deserves more liberal support at the hands of the benevolent. Last year the beds were occupied to their fullest capacity, no fewer than 1,092 patients being treated, while in the out-patient department there were no less than 39,871 consultations. At the convalescent home at Eastbourne, which is worked in conjunction with the hospital, 167 patients were received. For some years past the income of the hospital has failed to meet the expenditure, and at the present time the capital account has been trenched upon to the extent of nearly £12,000. A special effort is being made to raise this sum in order to place the institution on a satisfactory financial basis, and help is also urgently needed—particularly in the shape of annual subscriptions—towards meeting the yearly expenses of maintenance, which average about £10,000. Secretary-Superintendent, Mr. G. A. Cross, who is entitled to great credit for his zeal and success.

### MOUNT VERNON HOSPITAL.

PARTICULAR attention is drawn to the urgent need of completing the hospital at Mount Vernon, Hampstead, thereby increasing the accommodation from 100 to 140 beds. For many months past the duties of the medical staff have been of an exceptionally difficult and trying character. To be compelled to send away—not occasionally, but daily—applicants who are in every respect eligible for indoor treatment, and whose lives might thus be prolonged, is a terrible experience. But the difficulties do not end here. For those who are approved—and there are now nearly 250 such waiting their turn to be admitted—there remains the long and weary wait of at least twelve or fifteen weeks, during which time the disease which has attacked them pursues its course with such deadly effect that many who were not beyond hope of cure when approved, are hopelessly ill when they are received. It is to remedy these unsatisfactory conditions that help is required. Of the £20,000 needed to complete the hospital, £7,000 has yet to be subscribed.—Secretary, Mr. W. J. Morton, who deservedly enjoys the confidence of his board.

### HOSPITALS WITH FIFTY BEDS AND UPWARDS.

### VICTORIA HOSPITAL FOR CHILDREN,

CHELSEA, S.W.

THE committee are appealing for special donations to complete the new building fund; £10,000 is still required, and the hospital further seeks subscriptions to help it to carry on its work. Secretary, Mr. H. G. Evered.

### POPLAR HOSPITAL FOR ACCIDENTS,

BLACKWALL, E.

SITUATED amongst a teeming population of poor hard-working people in a district which may be called the "workshop" as well as the "port" of London, this hospital is doing an excellent work. The demands on the institution have of late years greatly increased, and consequently further subscriptions and donations are very necessary. Secretary, Lieut.-Colonel Feneran.

### ROYAL CHEST HOSPITAL,

CITY ROAD, E.C.

THE expenditure at this hospital exceeds £8,000, towards which there is an annual subscription list of some £2,300, and dividends amounting to £135. £7,000 are urgently needed to build and furnish a nurses' home. Donations will be gratefully acknowledged by the Secretary, Mr. Arthur T. Mays.

### QUEEN CHARLOTTE'S LYING-IN HOSPITAL.

THIS hospital received 1,284 patients into its wards last year, and, in addition, attended 1,026 patients at their own homes. The enlargement and renovation of the hospital have been completed, and much-needed additional accommodation is now available. Her Majesty Queen Alexandra has accepted the office of Patron, and has become an annual subscriber. A feature of the work during the past three years has been the admission as patients of the wives of soldiers and sailors sent on active service to South Africa. The expenditure amounts to £5,000 yearly, while the only reliable income amounts to £2,000. Contributions towards the deficit of £3,000 and towards the £7,000 still needed for the building fund are, therefore, earnestly solicited. Secretary, Mr. Arthur Watts.

### TOTTENHAM HOSPITAL.

THIS hospital has done surprisingly well since it was reconstituted a few years ago. The people of the district have taken an increasing and genuine interest in its progress, with the result that the beds have increased from 25 to between 70 and 80. Last year the number of in-patients under treatment was 713, and of out-patients 14,789. During the last three years the average gross annual income has been £4,923, and the average gross annual expenditure £4,719, a fact which points to careful administration. The most pressing requirements of this hospital are (1) additional income to the extent of £5,000 a year to maintain the beds in a new wing, the cost of erecting which we have confidence the London County Council will, if asked, consent to defray, so as to provide hospital accommodation for the new population of some 40,000 people which that Council is moving from the congested districts out to Tottenham; (2) £5,000 to enable certain structural alterations to be carried out, including an operation theatre and a couple of small wards for special cases. We look forward to the time in the near future when the Tottenham Hospital will be as well found, as extensive, and as well supported by the inhabitants of the districts it serves as the Great Northern Central Hospital is at Holloway.

### ALEXANDRA HOSPITAL FOR CHILDREN WITH HIP DISEASE.

THE necessity for this hospital for hip disease may best be shown by the fact that nearly three-fourths of its patients come from other London hospitals, the reason being that no general hospital can retain a patient for two or three years, as is not infrequently done in this hospital. £1,000 is urgently needed for maintenance. This hospital is beautifully kept, and it is without doubt the most pathetic of all our hospitals. We could wish that many wealthy and well-to-do women would give up an afternoon to visit this institution, for they would then certainly be moved to attach themselves permanently to its interests. Secretary, Mr. Stanley Smith.

### THE HOSPITAL FOR WOMEN,
SOHO SQUARE, W.

THERE are 64 beds, of which 19 are closed for want of funds. The committee very earnestly appeal for additional annual subscriptions and donations. Secretary, Mr. David Cannon. This is one of the most comfortable hospitals in London from the patients' point of view.

### WEST HAM AND EAST LONDON HOSPITAL.

THIS institution is doing a most useful work in one of the outlying and most congested districts of London. Of its 60 beds 46 during last year were daily occupied, and 2,126 in-patients and 32,176 out-patients were relieved. During the last three years the average gross annual income has been £6,332, and the average gross annual expenditure £5,696. Its most pressing requirements are—(1) more money in the shape of income; and (2) a further £5,000 to £10,000 to defray the cost of providing additional wards, an operation-theatre, and other necessary accommodation. We have always had good ground for entertaining a high opinion of the energy and devotion of the officials of this institution.

### HOSPITAL OF ST. JOHN AND ST. ELIZABETH.

THIS institution, which has recently been rebuilt in the Finchley Road, is a hospital which ought to have the active and liberal support of all Roman Catholics. It is most economically administered by a sisterhood, and anyone who will visit it will be pleased and satisfied. Of 59 beds at present equipped 51 were daily occupied during last year, and 95 in-patients were relieved. These figures point to the fact that a large number of relatively chronic cases are taken in who belong to the very poorest class, and who here find a comfortable home in the time of their greatest need. During the last three years the average gross annual income has been £2,882, and the average gross annual expenditure £2,539. These figures must be regarded as approximate, because the hospital has been rebuilt within the period named, and had the beds all been available and occupied the expenditure would largely have exceeded the available income. From all we hear of this hospital we have confidence in commending it to the liberal support of the charitable.

### NORTH EASTERN HOSPITAL FOR CHILDREN.

THIS institution suffers from the circumstance that it is situated at Hackney, and so never comes within the purview of the overwhelming majority of the larger contributors to hospitals. It does an excellent work amongst the very poor, and has a most energetic secretary in the person of Mr. T. Glenton Kerr. Out of 57 beds 47 during last year were daily occupied, and 722 in-patients and 19,116 out-patients were relieved. During the last three years the average gross annual income has been £6,206, and the average gross annual expenditure, £6,170. It is in need of £30,000 to enable the committee to complete and properly equip the new buildings, a portion of which are in course of erection. Anyone who desires to give wisely and directly in aid of the sick who are also very poor, will make a point of visiting this institution and contributing to the building fund.

### THE CHELSEA HOSPITAL FOR WOMEN,
FULHAM ROAD, S.W.

THIS hospital, which has 52 beds, was founded in 1871 for the reception and treatment of respectable poor women and gentlewomen in reduced circumstances.

The council appeal for donations and new annual subscriptions (their only reliable source of income) in order to meet increasing demands without incurring a heavy debt, and to pay off a mortgage of £4,000 on the hospital. There is a convalescent home at St. Leonards-on-Sea, which contains 22 beds, and is open to others than those who have passed through the hospital. Secretary, Mr. Herbert H. Jennings.

### ROYAL ORTHOPÆDIC HOSPITAL.

NOTWITHSTANDING that the work of this hospital is curtailed by the closing of two wards, its present income falls short of its requirements by £750, and its accumulated debt to bankers and tradesmen amounts to £2,000. Secretary, Mr. Tate S. Mansford.

### HOSPITALS WITH LESS THAN FIFTY BEDS.

### SAMARITAN FREE HOSPITAL FOR WOMEN AND CHILDREN.

THIS hospital is entirely free; it is dependent upon the charitable public for support; it needs £6,000 per annum. Funds wanted at once to meet overstanding liabilities. Committee appeal for bequests and donations towards a new endowment fund; £1,000 will endow and name a bed. £5,000 required to build a new outdoor department, increase the accommodation for nurses, and build a new operating theatre. Secretary, Mr. W. G. King.

### NORTH-WEST LONDON HOSPITAL.

WAS founded in 1878, and is the only institution of the kind in the north-west district. This hospital has great claims upon all thoughtful persons from the circumstance that Mr. George Herring, who is probably the most beneficent of the regular contributors to metropolitan hospitals, takes an abiding interest in its upkeep and maintenance. There is great need for a large sum to enable the committee to get out plans and erect a modern hospital, so that the inhabitants of the north-west districts of the metropolis may be adequately supplied with hospital accommodation. It has 53 beds, and last year there were 639 in-patients and 19,566 out-patients. Secretary, Mr. Alfred Craske.

### ST. SAVIOUR'S HOSPITAL

ASSISTS ladies of limited means. The number relieved last year was 143, and the cost of maintaining the hospital was £2,126.

### HAMPSTEAD HOSPITAL.

A NEW hospital is being built for the accommodation of the patients of this institution. The old buildings contain 32 beds, of which 24 were daily occupied during last year, when 379 in- and 3,965 out-patients were relieved. During the last three years the average gross annual income has been £2,838, and the average gross annual expenditure £2,950. These figures show wise economy of administration, and should command additional support. Its most pressing requirements are: (1) £30,000 to defray the cost of the new hospital now in course of erection, and (2) an increased income of at least £3,000 a year. Mr. R. A. Owthwaite, the honorary secretary, is one of the oldest and ablest of hospital secretaries, who has rendered immense service to the cause of hospitals generally in connection with the Hospital Sunday Fund. Mr. Owthwaite has quietly devoted the best years of his life to the promotion of the progressive development of metropolitan hospitals, and especially those which have been fortunate enough to secure his personal supervision and service. We mention these facts as a strong additional inducement to the wise to contribute liberally to the Hampstead Hospital.

### HOSPITAL FOR EPILEPSY AND PARALYSIS.

THIS hospital has been rebuilt upon a new site in Maida Vale, and will shortly be opened for the reception of patients. We have far too few beds for the accommodation of patients suffering from paralysis and epilepsy, and a contribution towards the £30,000 required to adequately finance this institution would be well laid out. Mr. H. Howgrave Graham, the secretary, has devoted many years of his life to the work of financing, under most difficult circumstances, this hospital, and we hope that many important charitable people will visit the hospital and identify themselves with its work.

### MILLER HOSPITAL AND ROYAL KENT DISPENSARY.

THE Miller Hospital was the first general hospital erected in this country with circular wards. It is a comfortable little place, ministering to the needs of a large population in Greenwich and the neighbourhood. For some reason, which it is difficult to account for, it has never attracted the support which it is entitled to receive from the inhabitants of Blackheath and the district which it serves. Its most pressing requirements are: (1) £20,000 to extend the hospital and refloor the wards; (2) additional accommodation for the nursing staff; and (3) £3,000 a year more income. The importance of the work it does will be seen from the statement that during last year 352 in-patients and 17,369 out-patients were relieved. Mr. James Marks, the secretary, is an old and faithful servant of the institution whom it is always a pleasure to see when visiting the hospital.

### GROSVENOR HOSPITAL FOR WOMEN AND CHILDREN,

VINCENT SQUARE, WESTMINSTER, S.W.

THIS hospital, formerly inadequate and ill-contrived, has been rebuilt with modern improvements. Increased support is needed, two wards being still closed for want of funds. Secretary, Mr. W. J. Davidson. There is a deficiency of £400 on the year's working. An increase of £1,000 a year in the income is required to enable all the unoccupied beds and wards to be reopened, and Lady Aberdare has promised to give £1,000 as the first of ten similar amounts to complete the sum of £10,000 required for building and other purposes. It is an economically administered, pleasant, quiet little hospital, and deserves the support of the generous, and especially of those who are interested in women and children.

### CHARITY BY DEPUTY.

MOST of our charity is done by deputy. Here and there we may take a little personal trouble for some person or thing that excites our sympathy; but for the most part we salve our conscience by giving a subscription to some society, and never even ask ourselves what is done with our money. It may be wisely spent, or it may be squandered; we hardly know, and it might truthfully be said, we hardly care.

In defence, we may truthfully urge that if we are to be charitable at all, we must use other people's heads and other people's hands to distribute our beneficence. We have our living to earn, and the utmost we can do is to give a part of our earnings to the poor and needy. Yet a hard-working man, or a careful woman, hates waste; however generous they may be with their gifts, they do not want to see them abused, and that there is much abuse of charity no one will doubt. To define this abuse as simply waste is to speak too kindly of it. If in almost every case, money that does not do good does harm, in the case of charity it is almost invariably so. If of the money given by the public to help the sick and the needy a great proportion goes in the cost of collection and in office expenses, we may be quite sure that the kind-hearted and easy-going donors, instead of doing the good they hope for, are simply maintaining a more or less useless host of middlemen.

That the society, or whatever its name may be, for relieving distress of one kind or another is a convenience cannot be denied; but it is very often a fatal convenience. Even when we begin by a real and personal interest for some sick, or poor, or lonely creature, we feel that we have done all that can be expected of us—or even that our conscience expects—when we hand over the case to the society which professes to deal with such ills, and give a subscription to its funds. That personal interest, personal affection, extends beyond this, enters into the minds of comparatively few. The superintendents of institutions for the incurable and the insane admit, regretfully, that many of those under their care are hardly ever visited. If you were to seek out the friends of these patients and ask them why they so neglect their annuitants, who are, perhaps, their kinsfolk, they would be sure to plead pressure of business and perhaps add that a visit is so painful. The plea may be true; but, on the other side, what can be more painful to the invalid than to feel himself thus thrust in a corner, fed and clothed because law and custom forbid those more summary methods by which savages get rid of their aged and infirm, but regarded by his civilised friends as simply a burden and an annoyance, just as he might be were he a member of some wandering tribe that earned a precarious living by prowess in the chase. It may be said that the ailing and, above all, the insane, are not so sensitive, but those who are always with them know that in many cases they feel most keenly the neglect from which they suffer.

But charity demands more of affection than even affection's self. If we are really to help the suffering, whether they are bound to us by the ties of love and kinship or only by those of charity, we must help them wisely. Simply to put them into almshouse, or infirmary, or asylum may be to do no more than to give the proverbial organ-grinder a penny to go away. We do something to relieve our own feelings; above all to get rid of a painful object, and inquire very little if this is the best thing we can do for those in whom we profess ourselves interested. At this point, charity by deputy fails completely. On this weakness thrive a multitude of so-called homes, retreats, and sanatoria, which are neither more nor less than the rubbish heaps of humanity. The feeble and useless members of society are cast away there, and their own kinsfolk suppress the fact of their existence as carefully as if they were incarcerated in Dartmoor. That public sentiment is beginning to awake to the fact that you do not fulfil all your duty to a human being by saving him from dying in the streets is true, but comparatively few of those who contribute of their abundance to charities realise that their duty to their fellows does not end there, but only begins. Those who encourage the patients in hospitals for incurables to do little pieces of work are fulfilling the most Christlike charity, in making these unfortunates realise that they have still some use and value in the world.

But seeing that they are not entirely helpless logs, why should they be in the hospitals at all? Have they no friends or kindred willing to take care of them? It may be pleaded that their friends, however willing they may be to keep them, cannot afford to do so. Then it becomes a question whether it would not be better to expend the money which it costs to keep them in a more or less palatial building, maintained at very considerable expense, in giving such a pension as would enable them to be kept at home. It is not wholesome for anyone to see none but those exactly like themselves. It is not wholesome, not to say exhilarating, for incurable patients to see none but other incurables, to have nothing to talk about but their respective symptoms, and the care or neglect which they receive from nurses and doctors. Some of the saddest weaknesses of humanity come to the surface in such an environment, a very rivalry of selfishness grows up, from which the monotony of institutional life gives to distraction. And if the patient is selfish, the kinsfolk are more so. The visits to the invalid are felt to be only a painful duty, until at last the sense of duty is left out and only the sense of pain remains, and is shirked, as pain always is. Would it not be better in most cases to leave the invalid at home, give such help as will prevent his being a pecuniary burden to his relatives; but leave him to fulfil the mission which we may infer God meant when He afflicted him—to draw out the feelings of tenderness and pity, which his afflictions should arouse in those around him, and perhaps to preach silently from a sickbed those lessons of faith and patience of which an eager and selfish world stands too much in need?

To remove the sick-poor who are suffering from acute diseases to hospitals where their chances of recovery will be better than in their homes, is reasonable and wise, but it is a very different thing to remove them from these homes, poor as they may be, to linger out the remnant of their days in an institution. Dante's gloomy motto, "All hope abandon, ye who enter here," might truthfully be written above many of these places.

The fashion of almshouses is happily dying out. They were pretty and picturesque, no doubt, but charity is something more than landscape painting. Strange as it may seem, it is often a cruel kindness to take old people who have spent their lives in the back street of a busy town and plant them down in the peaceful country, however picturesque it may be. They will be happier in their garrets in the slums, where at least they could see old cronies, receive visits from children and grandchildren, and keep in touch with the interests of their active days.

Without multiplying instances, it is easy to make it clear that much of our charity does less than it is intended to do, and that when, as is often said, the poor are ungrateful, their ingratitude is comprehensible. Do we not ourselves often feel thankless to the donor of a gift, which, as we say, represents only so much money; while our eyes fill, and our hearts glow, at the sight of the far less costly trifle which yet happens to be something we need, or something for which we have expressed a wish, and which shows that the friend who gave it took trouble to gratify us. Unless a similar spirit animates our charity we have no right to complain of ingratitude. If it is more blessed to give than receive, it must be because the giving is no mere casting of money into the treasury, but a careful, and even painful choice of what it is best to do for those whom we would help. This usually means more than money; it means time and thought and personal service, but without these things, our charities may be as useless and even harmful as the lavish alms of mediæval convents, which, for every case in which they helped real distress, bred sturdy beggars in a score. Personal service and careful consideration of the best interests of those we help is the end of all true charity. Our motto must be—

"Not what we give, but what we share,
For the gift without the giver is bare."

### THE GENERAL CHARITIES.

In addition to the general and special hospitals there are a number of general charities which at this season of the year merit the liberal support of the public. It is most essential, however, that great discrimination should be exercised in responding to the multitudinous appeals which are issued, and as a guide to our readers we select a few which have given satisfactory proof, by good management and careful expenditure, that they are thoroughly deserving.

### ASSOCIATION FOR THE ORAL INSTRUCTION OF THE DEAF AND DUMB.

This association is entitled to the credit of being the first to publicly introduce into the United Kingdom the German, or pure oral system for teaching deaf and so-called dumb children to speak *viva voce* and to understand the spoken words of others by lip-reading. The expenses, which are very heavy, are met by voluntary contributions and fees, which fall short of the amount imperatively needed to the extent of about £600 per annum. The Director is Mr. William Van Praagh, 11 Fitzroy Square, W.

### IRISH DISTRESSED LADIES' FUND.

Relief is given by this society independently of any question of politics or religion. It also, if possible, finds employment for those who are able to work, affords pecuniary assistance to the aged and infirm, and pays for the education of children. There are 78 pensioners on its books this year, mostly resident in Ireland, and 98 ladies are helped to earn their living through the work depôts in London and Dublin. The Secretary is General W. M. Lees, 411 Oxford Street, W.

### LONDON SCHOOLS DINNER ASSOCIATION.

This is an organisation which, in view of the attempts that are constantly made to throw upon the rates burdens that charity is able to bear, should commend itself to many. It makes grants to provide cheap, or free, meals for necessitous children attending the London public elementary schools, and has an average income of about £1,200. More, however, is urgently needed so as to place the Association in the position of being able to make immediate grants in cases of proved necessity which are recommended by responsible local authorities. The Hon. Treasurer is Lord Kinnaird, and the Secretary Mr. T. A. Spalding, 117 School Board Offices, Thames Embankment, E.C.

### THE MARY WARDELL CONVALESCENT HOME FOR SCARLET FEVER.

This is the only Home for Convalescents from scarlet fever, which is always more or less rife in the Metropolis. There is urgent need for £2,000 to repay a loan which had to be raised for the purpose of defraying the cost of relaying the entire drainage system to meet the requirements of the District Council and to clear off other debts. The repayment of the loan is being pressed for, and the Home is absolutely without funds. Donations and subscriptions may be sent to Miss Mary Wardell at the Home, Stanmore, Middlesex, or to Messrs. Barclay and Co., Limited, 1 Pall Mall East, S.W.

### ROYAL ASSOCIATION IN AID OF THE DEAF AND DUMB.

This society sends visitors to deaf and dumb persons in their own homes, and assists those in sickness and distress. During the past year 3,145

visits were paid to the deaf and dumb, and 1,121 to employers and others on their behalf; 200 were relieved and 57 provided with work and apprenticed. An increased reliable income is required to maintain and extend the present work. The Secretary is Mr. Thomas Cox, and the offices of the Association are in Oxford Street, W.

### SHIPWRECKED FISHERMEN AND MARINERS' ROYAL BENEVOLENT SOCIETY.

A PROMINENT feature of the work of this society is to grant relief to shipwrecked persons, to widows and orphans of members and others, and to destitute seamen. Grants are also given towards meeting the loss of clothes or boats, and help is afforded in old age or in case of infirmity. Nearly £18,000 was expended in relief in 1901 to 9,373 persons. The Secretary is Mr. Gerald E. Maude, 26 Suffolk Street, Pall Mall East, S.W.

### ROYAL BLIND PENSION SOCIETY.

THIS society provides pensions by monthly instalments for blind people. The number thus assisted is 1,087. Further contributions are greatly needed, particularly annual contributions, in order to enable it to help 200 eligible candidates, who are seeking relief from the society. Secretary, Mr. W. Terry, 237 Southwark Bridge Road, S.E.

### BOURNEMOUTH NATIONAL SANATORIUM FOR CONSUMPTION AND DISEASES OF THE CHEST.

PATIENTS in humble circumstances who are convalescent, who require further medical treatment and change of air, or who are suffering from an incipient form of the disease, are cases for the relief of which this institution was founded. The "open-air" treatment is carried out completely in all suitable cases. There are at present only beds for 31 men and 31 women. Owing to this limited accommodation, patients who require speedy relief have often to wait many weeks before they can possibly be received. In some cases, when their turn comes for admission, it is *too late* for them to receive benefit by treatment at the Institution. To meet the rapidly increasing demands, building extensions are being provided to add 10 beds, and for this purpose about £800 is still required. The committee earnestly solicit liberal support, as this national sanatorium is almost entirely dependent on voluntary contributions. Secretary, Mr. A. G. A. Major.

### ORPHAN WORKING SCHOOL,

HAVERSTOCK HILL, N.W., AND HORNSEY RISE, N. OFFICES, 73 CHEAPSIDE, E.C.

A NATIONAL and undenominational institution which maintains 500 children, varying in age from infancy to fourteen or (in special cases) fifteen years. It is greatly in want of funds at the present time. Appeal is made specially for new annual subscriptions, the normal income of the charity being more than £2,000 below what is needed to meet the expenditure. Secretary, Mr. Alexander Grant.

### LONDON ORPHAN ASYLUM.

THIS institution during the last 90 years has benefited nearly 7,000 children of professional men, agriculturists, trade and mercantile clerks, and others. The orphanage is more catholic than its title would indicate, for it receives children from all parts of the country, and even from the colonies. The children are well and carefully trained, the girls on leaving becoming Board School teachers or civil servants, and the boys being provided with situations with board and lodging, or, where friends can admit them into their families, with employment in mercantile houses. The most pressing requirements of this institution are £14,000 a year in annual subscriptions and donations, and £3,500 to free the institution from debt. The Prince of Wales, at the festival dinner in April, declared that he could not exaggerate the favourable impression left on his mind by a visit to the institution. He felt that the ideas of the founders had been more than fulfilled, and everywhere there was evidence of the care and solicitude shown for the moral, intellectual, and physical well-being of the children. The Secretary is Mr. H. C. Armiger, 21 Great St. Helen's, London, E.C.

### THE CHILDREN'S HOME AND ORPHANAGE,

BONNOR ROAD, LONDON, N.E.

THIS orphanage has branches in many places, and 1,2[illegible]0 boys and girls are at present dependent upon it. Miss Fowler, of Liverpool, has promised £20,000 to open one such branch in the North of England which will provide accommodation for 300 children, entailing an expenditure of several thousands a year. The most pressing requirements are £2,000 per annum additional income, and £4,000 to defray the cost of erecting a cripple and convalescent home at the seaside in order to secure conditional gifts of £500 from Mr. Joshua Locke and Mr. John Cory respectively.

### NATIONAL ORPHAN HOME,

HAM COMMON.

THIS orphanage is under the management of a committee, of which the Vicar of Ham is an active member. Fifty-five children were in the orphanage during last year, and the school has earned the maximum grants from the Education Department. This institution is badly in want of additional annual subscriptions to give it a position of permanence and stability. The secretary is Mr. George C. Hobson the Home, Ham Common.

